# From clinics to (cow)mics: a reproductive journey

**DOI:** 10.21451/1984-3143-AR2018-0076

**Published:** 2018-08-17

**Authors:** Patrice Humblot

**Affiliations:** 1 Division of Reproduction, Department of Clinical Sciences, SLU, Uppsala, Sweden.

**Keywords:** cattle, genomics, reproductive phenotypes, reproductive technologies

## Abstract

This manuscript describes the different topics I have been involved in the fields of reproductive physiology and embryo biotechnologies with attempts to address practical issues raised mainly by the breeding industry. The journey started with phenotyping work in the field of reproductive physio-pathology. Other issues were related to the optimization of reproductive biotechnologies to favorize genetic selection. The implementation of genomic selection raised opportunities to develop the use embryo biotechnologies and showed the interest of combining them in the case of embryo genotyping. There is still a need to refine phenotyping for reproductive traits especially for the identification of markers of uterine dysfunction. It is believed that new knowledge generated by combining different molecular approaches will be the source of applications that may benefit AI practice and embryo technologies.

## Introduction

### Warning!

Working most of my life for the breeding industry had two major consequences. This led to develop research based on application driven approaches. In addition, although working mainly in the field of animal reproduction, I have been almost permanently at the border of different domains, endocrinology at first, embryology and embryo technologies, genetic selection and more recently reproductive genomics. I started with clinical medicine, dealing with reproductive problems in high producing dairy cows and approach today the mechanisms underlying the development of inflammation and resilience to stress, using cow, dog and cat endometrium as models.

The following text is an attempt to describe the context in the field of reproduction at the beginning of my working life, the major developments in reproductive physiology, veterinary medicine and genetic selection, I have been witness too and their promising applications followed or not by real development. The environment of present research providing extremely powerful tools, especially for genomics, stresses out the need for Bioinformatics to integrate information when approaching reproductive physiology or diseases with concepts referring to precision medicine.

Hence, this text should be seen as just an overview produced by a “generalist” who approached too many topics. Despite the associated limitations, I hope that the description of existing gaps in knowledge and/or some of the perspectives drawn from it may be the source of research ideas for future adventurers discovering by mistake this text on a dusty shelve.

## AI and the birth of phenotyping for fertility

### Clinical approaches for the control of fertility and oestrus synchronization

I joined the world of Artificial Insemination (AI) in 1977, soon after the end of its golden age. Following a rapid growth after the creation of the first French AI centre in 1946 the number of AI’s reached a plateau in the 70’s and then started to decrease, due to the decrease in cow numbers associated with the increase in cow productivity. This technique is still widely used in France with a total of 7 millions of AI’s in 2017 ( Grimard *et al*., 2018 ) and represents the major way of reproduction in dairy cows (in 2017, 80% of calves issued from AI) whereas it’s development has been limited in beef cows (only 13% of calves issued from AI). Due to genetic selection oriented essentially to improve milk traits, fertility after AI decreased regularly between the 70’s and year 2000 ( Barbat *et al*., 2010 ). The above trend was not specific to France, and was observed in all dairy producing countries ( Royal *et al*., 2000 ; Lucy, 2001 ; Bousquet *et al*., 2004 ). The need for a better characterization of reproduction and treatment of reproductive disorders emerged from this situation. In the 70’s major progresses in the mechanisms regulating corpus luteum and pituitary function and the commercialization of hormones such as PGF2α and GnRH combined with the development of accessible progesterone Radio-Immuno assays offered new opportunities for the treatment of reproductive disorders ( Thibier *et al*., 1977 ; Humblot and Thibier 1980 , 1981 ). Progesterone in plasma then milk allowed the characterization of ovarian activity and the different types of postpartum reproductive disorders. Achieving this was “the birth of precise reproductive phenotypes” (As Mr Jourdain in “Le Bourgeois gentilhomme” [Molière] who did speak “prose” without knowing it, we were establishing the first reproductive phenotypes…) and gave opportunities to develop targeted treatment protocols depending on ovarian cyclicity ( Humblot and Thibier 1981 , Thibier *et al*., 1985 ). These studies were followed by the wave of systematic synchronisation treatments followed by fixed time AI(s) known today as FTAIs (see for review Sartori *et al*., 2016 ). All these first steps together with the development of efficient protocols to synchronize oestrus in dairy and beef cattle ( Chupin *et al*., 1974 ; Deletang, 1975 ; Grimard *et al*., 1995 ; Humblot *et al*., 1996 ) were crucial for the subsequent emergence of embryo based technologies.

### Characterization of reproductive disorders Post AI / embryo mortality:

In ruminants, the access to progesterone assays, the discovery of pregnancy specific proteins from the conceptus ( Martal *et al*., 1979 ; Thatcher *et al*., 1989 ) and later produced by placental cells ( Butler *et al*.,1982 ; Sasser *et al*.,1986 ; Beckers *et al*., 1999 ; Perenyi *et al*., 2002) allowed deciphering the mechanisms by which pregnancy was maintained or leading to embryo mortality. In the cow, the consequences of embryonic losses on luteal function were determined ( Northey and French, 1980 ; Humblot and Dalla Porta, 1984 ) showing that contrary to later losses, embryonic mortality before day 14 post-AI do not induce any change in oestrus cycle length. This information associated with the characterization of the Pregnancy Associated Glycoproteins (PSPB/PAGs) profiles were the basis to determine the relative weight of early and late embryonic losses ( Humblot *et al*., 1988 ; Humblot 2001 ). The results, obtained from thousands of cows (both dairy and beef breeds) showing the higher frequencies of very early losses (either non fertilization or early embryonic mortality) when compared to later losses were further confirmed with other methods and in a different environment and breeds ( Diskin *et al*., 2006 ). In addition, PAG measurements when repeated sequentially allowed the precise characterisation of the time of embryo death or later abortions. While revealing the strong gap between the time at which embryo death occurred and clinical abortion (reaching often 2 months or more) they represented a much better source of information to identify the causes of pregnancy failures and were the source of more precise phenotypes for pregnancy failures ( Dobson *et al*., 1993 ; Wallace *et al*., 1997 ; Humblot, 2001 ). More recently, new systems have been successfully developed for assaying PAGs in cow blood or milk ( Ricci *et al*., 2015 ). At the same time the PAGs family has been enriched with some new members allowing their measurement at an earlier stage of pregnancy ( Touzard *et al*., 2013 ), but today there is still a need for a specific and reliable marker of “non pregnancy” which being predictive of return in oestrus, would allow the planning of a new AI.

### The bull as a major source of variation of fertility

When the decrease in fertility following AI was ascertain, investigating differences in fertility between AI bulls became a concern. At the beginning of the 80’s, we analysed data from AI centres reputed for their proper management of information, gathering at this occasion the results of several millions of AIs and the available sources of variation which represented huge data sets. The procedures for the transfer of data, their validation and statistical analysis had to be customized and computing time and resources appeared as limitations. Although the bull factor was found significant, the results revealed that differences originated from a few extreme individuals representing less than 10% of the population (either with a very high or a very low fertility). The sequential analysis of non return (absence of oestrus following AI, being predictive of pregnancy) rates recorded at different times after AI did show that the bull used for AI influenced almost exclusively non fertilization or early embryonic mortality (before 14 days of pregnancy in the cow), subsequent impacts on fertility (late embryonic mortality or abortions) being very marginal ( Humblot *et al*., 1991 ). These results were further confirmed in one field trial where fertility phenotypes were defined with more precision from few thousands of AIs ( Grimard *et al*., 2006 ). Beyond the results obtained, the above work was an excellent opportunity to develop a fruitful collaboration with bioinformaticians and biostatisticians. This challenging experience, as a young reproductive physiologist, strongly influenced my education and way to approach research.

### Changes in AI practices and sperm processing; consequences for reproductive performances and the environment

In cattle, the landscape of AI has changed considerably since the 70’s. The changes are essentially linked to increased herd size, improved automatization and recordings associated to a relative reduction in man-power. This combination was not necessarily favourable to fertility as AI success is still related to the quality of heat detection in the absence of FTAIs. Two major changes in AI practice and technology occurred during the past decades ( Grimard *et al*., 2018 ). i) The number of AI performed by farmers instead of specialized AI technicians increases regularly especially in herds >100 cows (for instance +12% in France between 2015 and 2016). The impact of this practice on fertility is difficult to evaluate as non-return rates are evaluated from records deviating from the usual standards. ii) Semen sexing became a commercial reality with patent advantages for individual farmers and breeding companies. There is still no alternative to flow cytometry and related logistics ( Galli, 2017 ). Today, there is still differences in fertility (from 8 to 15% lower) in cows and even heifers following AI’s performed with sexed and conventional semen ( Le Mezec, 2018 cf review from Grimard *et al*., 2018 ) showing that the unfavourable consequences of sperm processing through flow cytometry are not fully controlled.

The advantages of encapsulation of sperm giving more flexibility in timing of AI have been put forward for a while ( Ghidoni *et al*., 2008 ), and improvements of sperm quality have been reported in an *in vitro* study ( Alm-Kristiansen *et al*., 2018 ). However so far field results are not so demonstrative ( Standerholen *et al*., 2015 ). Other issues relate to the environmental impact of adding antibiotics during sperm processing. Due to the large amounts of extenders to be prepared, inducing antibiotic resistance may be of critical importance especially in the pig ( Morrell 2016 ; Morrell and Humblot, 2016 ). With this perspective, all alternative solutions lowering the potential impact of sperm handling on the environment would be most useful.

For each of these fields related to semen processing and AI practice, new technological developments are awaited to improve fertility results while limiting at the same time possible impact of AI technology on the environment. In the near future, profit may be taken from the evaluation of seminal plasma (SP). Effectively, protein patterns in SP, which are representative of individuals, have been related to resistance to freezing, survival of sperm in the genital tract ( Soleilhavoup *et al*., 2014 ; Rickard *et al*., 2015 ), fertility ( Morrell *et al*., 2018 ) and also to be involved in immune-tolerance mechanisms which may be of importance especially for the success of implantation ( Robertson, 2005 ).

### Changes in genetic selection objectives and consequences for reproductive performances

In the seventies inheritance of fertility and its relationship with dairy production was already a concern ( Foote, 1970 ; Maijala, 1976 ). However, reproductive performances, still acceptable by this time declined steadily and even more during and after the 80’s ( Royal *et al*., 2000 ; Barbat *et al*., 2010 ). Following studies developed initially in Nordic countries, mostly Sweden ( Maijala, 1976 ), studies on the heritability of fertility traits ( Humblot and Denis, 1986 ) suggested that genetic selection for milk yield could be partly responsible for the decline in reproductive performance. These results were confirmed and much documented from further studies demonstrating strong negative genetic links between milk production traits and reproductive traits in all French dairy breeds ( Boichard and Manfredi, 1994 ; Ducrocq *et al*., 2008 ). This led to develop a genetic evaluation based on fertility and other functional traits, which was routinely used in France since year 2000 and helped to adjust breed selection objectives.

In most European countries, genomic selection has now been implemented for about 10 years. It is well established that genomic selection especially due to increased precision is much more efficient than former selection from quantitative genetics to orientate favourably reproductive traits or other traits with low heritability ( Barbat *et al*., 2010 ). Considering selection objectives which are more balanced than in the past, this could lower considerably the decrease in reproductive performance observed these last decades in most dairy breeds or even lead to some recovery ( Barbat *et al*., 2010 , Le Mezec, 2017 ). However, in countries where the use of FTIA protocols is very frequent, there is also a risk to select cows for their responsiveness to oestrus synchronization treatments instead of selecting for more physiological fertility traits ( Lucy, 2001 ). Despite a more balanced selection, dairy farmers will have to deal with individuals producing more and more ( Britt *et al*., 2018 ) and all problems related with high production are far from being solved. Although improvements in diets and management of feeding takes place there is still strong individual variations in the way the cows are dealing with the metabolic challenge they are faced too ( Bedere *et al*., 2017 ; Mellouk *et al*., 2017 ; Ntallaris *et al*., 2017 ). Responses to lactation and feeding are associated to huge differences between individuals in changes in Negative Energy Balance, body condition and fat mobilization ( Mellouk *et al*., 2017 ; Ntallaris *et al*., 2017 ). Effects of energy restriction on reproductive performance due to excessive fat mobilization can be even more pronounced in suckled beef cows ( Grimard *et al*., 1995 , 1997 ). Understanding these issues may help to find solutions to lower the amplitude of these changes during the postpartum period. This may lead in turn to a better control of the reestablishment of ovarian activity and overall reproductive performance. In this perspective, the impacts of fat mobilization on inflammatory processes and sensitivity to post-partum diseases such as endometritis ( Wathes *et al*., 2009 ; Valour *et al*., 2013 ) are major issues but practical diagnostic tools are still missing. Such tools are needed to diagnose cows with sub-clinical inflammation, decide if AI is appropriate or not and define alternative therapies. In addition, such markers will give the basis for new phenotypes to be used in future selection programs with the objective to produce more robust animals, not only for reproductive traits but also for resistance to diseases.

## Embryo technologies

The development of embryo based biotechnologies started in the 70’s. The use of MOET’s (Multiple Ovulation Embryo Transfer) programs became very popular and the proportion of bulls favourably tested issued from embryo transfer and used massively as AI sires, increased very quickly to reach about 90% before the development of IVF-IVP (*in vitro *fertilization- *in vitro* production) occurring in the 90’s.

The technical developments of *in vivo* embryo transfer, which took place during these 20 years have been reviewed by Ponsart et al., 2004 . Superovulation protocols included initially the use of eCG (equine Chorionic Gonadotrophin), which has been replaced successfully by FSH in the 80’s ( Nibart and Humblot, 1997a ). Crucial improvements occurred with the use of non-surgical techniques for collection and subsequent transfer of embryos together with the optimization of freezing protocols ultimately allowing routine use of direct embryo transfer which was thus performed as an ordinary AI. The development of procedures insuring the quality of field work and the safety of conditioning fresh and frozen embryos made embryo transfer the safest way to exchange genes as reviewed by Thibier 2001 , 2011. I contributed marginally to these first improvements, the corresponding work from our group being performed mainly by M Nibart who was coordinating the activities of embryo transfer technicians in France and established first strong connections with Brasil ( Nibart *et al*., 1997 ) where the success of the technique became exponential. In commercial groups in France, as well as in other countries in Europe mean pregnancy rate close to 60% were easily achieved after on-farm non surgical transfer of single fresh embryos ( Nibart and Humblot, 1997b ; Ponsart *et al*., 2004 ). Embryo sexing became considered, to better target the use of embryo transfer either for the benefit of the farmer who wanted new female calves of a high genetic merit or to provide male calves as future sire candidates for breeding companies ( Thibier and Nibart, 1995 ). The success of embryo development following biopsy and achieving pregnancies from frozen and biopsied embryos became critical ( Lopes *et al*., 2001 ). Pregnancy rates over 60% were rapidly obtained by different groups following the transfer on farm of fresh biopsied *in vivo* produced embryos ( Lacaze *et al*., 2008 ; Ponsart *et al*., 2008 ) and later on similar percentages were reported following use of frozen biopsied embryos either on farm or in station ( Gonzalez *et al*., 2008 ).

IVF-IVP and subsequent embryo based technologies such as cloning have been reviewed very nicely and extensively by C. Galli (AETE pioneer award 2017). As it would be inappropriate and vain to develop the matter with a similar approach, we will focus here on the practical issues we did try to address with the group in the Research and Development department of UNCEIA (Union Nationale des Coopératives d’Insémination Animale). The advantages and some of the questions raised by the use of these technologies and emerging ones in selection schemes especially in relation with genetic variability will be discussed.

### Improving the quality of oocytes and embryos

While postpartum dairy cows meet a more or less pronounced status of negative energy balance (NEB), investigations performed in donor cow and heifers revealed that these do not usually suffer from energy deficit ( Humblot *et al*., 1998 ). On the contrary, embryo donors were very frequently overfed and present high concentrations of glucose and insulin associated to a high Body Condition Score (BCS). Due to positive effects on follicular growth, these characteristics may be favourable to the superovulatory response but not necessarily to fertilization and early embryo survival. In donor cows with high BCS, the number of unfertilized oocytes was increased ( Humblot *et al*., 1998 ). Superovulated dairy heifers submitted to a high growth rate presented high concentrations of insulin ( Freret *et al*., 2004 ) and blastocyst development following repeated OPU (Ovum Pick up) and IVF was decreased when compared to restricted ones ( Freret *et al*., 2006 ). This led to the concept that a transient increase of energy could be favourable to follicular growth and superovulatory response whereas constant exposure to high energy may affect negatively fertilization and early embryonic development ( Humblot *et al*., 2008 ; Garnsworthy *et al*., 2009 ).

It was confirmed later on, that exposure of restricted donor heifers to a transient increase in energy brought by propylene glycol which increased insulin levels, improved the superovulatory response and the production of high quality embryos ( Gamarra *et al*., 2015 ). Although the full mechanisms by which such effects are induced is still to be deciphered, the favourable changes observed could be related to restoration of critical gene expression of the IGF system in follicles associated to epigenetic effects in blastocysts ( Gamarra *et al*., 2018 ). There is still issues to be solved while making the above diets attractive for donors and their use practical. If successful, they may be applied also more extensively in dairy or beef cows for which energy supply is often a limitation ( Grimard *et al*., 1995 , 1997 ). The positive effects of improved diets could be used to optimize the results of MOETs or OPU-IVP programs through more “personalized approaches” when implementing superovulation protocols or even before AI. Similarly, the measurement of anti-Müllerian hormone (AMH) which helps to predict an animal's response to superovulation ( Rico *et al*., 2009 ; Mossa *et al*., 2017 ), may be used to individualize treatment protocols. This is probably more promising than implementing selection on this phenotype that would result in a drastic reduction of families in selection schemes detrimental to genetic variability.

### Embryo technologies and selection

At the same time we tried to improve reproductive technologies and control better the factors influencing the success of superovulation, *in vivo* and *vitro* production, pregnancy rates after embryo transfer as fresh or frozen, work was done on the concept of assembling different techniques for the sake of genetic selection and later on in the emerging context of genomic selection. These efforts were both, technically and politically driven. Our work was supported by breeding companies, thus, demonstrating the advantages of the different embryo based biotechnologies for selection purposes was a major concern mixed with the necessity of making embryo based techniques economically sustainable. Although being reproductive physiologists, our “*Credo*“ was; Genetic progress:

$$\;\frac{\Delta g\;=\;\left(selection\;pressure\;x\;precision\;x\;genetic\;variability\right)}{Generation\;interval}$$

We had to consider how reproductive biotechnologies could serve each of the terms of this basic equation. This was not so simple as for instance selection pressure and the possible resulting genetic variability of a given trait are antagonistic. Also, as mentioned before, due to negative genetic correlations, selecting exclusively for a given trait would be detrimental to other traits in a very “efficient” way (this has been demonstrated from the example of milk production and reproductive traits). The practical cost of the techniques was an additional parameter conditioning our activities and the respective development of each embryo based technologies. Due to this combination of constraints, the different techniques were more or less affected by the evolution of genetic selection and the environment of the milk market. In France and more generally in Europe, AI and *in vivo* embryo transfer were well implanted, considered as robust and not too expensive under well established routines. Implemented by AI technicians/or specialized ones their application by the breeding companies was not put into question. On the contrary, although significant improvements in embryo production related to oocyte maturation ( Humblot *et al*., 2005 ; Lequarre *et al*., 2005 ), culture systems ( Menck *et al*., 1997 ; Guyader Joly *et al*., 1998; Holm *et al*., 1999 , 2002 ) and embryo freezing ( Vajta *et al*., 1997 , 1999 ; Guyader Joly *et al*., 1999; Diez *et al*., 2001 ) have been achieved, IVF-IVP technologies were chronically and sometimes acutely seen as expensive, not always reliable or not efficient enough. In the context of genetic schemes, using males of a high genetic merit, not necessarily among the most fertile ones was mandatory. Despite efforts were made to customize the *in vitro* production system, especially fertilization steps (Marquant Le Guienne *et al*., 1990; Marquant Le Guienne and Humblot, 1998), the direct effect of the bull on fertilization and early development rates as evoked above in the context of AI, represented often (and still represents) an additional limitation for the production of viable embryos. In addition, the need for consistent investments, both in terms of facilities (laboratory and station) and personnel, made them regularly criticized. Despite strong advantages especially in terms of generation interval and genetic variability were seen ( Humblot *et al*., 2010 ; Humblot, 2011 ) they did not balanced sufficiently the above limitations in the hands of European “genetic drivers”. However, the economical situation and bases for marketing genetics were totally different in other parts of the world, especially South America and most particularly Brazil, where the growth of IVF-IVP techniques became exponential at the same time these were confronted to limitations in their development in Europe (the number of transfers with *in vitro* produced embryos were reduced by -33% between years 2003 and 2004; Lonergan, 2004 ; Merton, 2005 ).

Things started to change and a new era opened for embryo based biotechnologies with the emergence of the first generation of genomic selection. In the bovine species, the discovery of DNA regions where polymorphism was associated with phenotypic performance for traits of interest (QTL; Quantitative Trait Loci) was at the origin of the present revolution in the selection process. From 2000 to 2005 a few QTL of interest were available and the idea emerged to genotype embryos for those markers before transferring them with the main objective to increase selection pressure. This was the birth of the programme “TYPAGENAE” in which we planned to combine different embryo biotechnologies to perform genotyping on embryonic material ( Le Bourhis *et al*., 2008 , 2010 ; Humblot *et al*., 2010 ; Humblot, 2011 ). Due to the very limited amount of biological material available from the biopsy, it was planned to use biopsy culture techniques and cloning of blastomeres to satisfy the DNA requirements for typing. Although possible, such procedures where quite heavy to set up for a routine use and fortunately very quick improvements in DNA typing techniques allow bypass these steps. All the work previously done to improve the freezability of biopsed *in vivo* or *in vitro* produced blastocysts was valorized in this application (Guyader Joly *et al*., 2008). By the time the programme was initiated some advantages were found in terms of genetic progress ( Humblot *et al*., 2010 ). Surprisingly we observed that the efficiency of the technical steps were not among the major variables influencing genetic gain. The need for a high selection pressure and economical factors such as the price of heifers had more weight than any of the reproductive steps involved in the process. However this was found in the context of selection for a single trait. As selection for multiple trait is much more demanding in terms of genetic resources, it is likely that the efficiency of reproductive techniques would be more important in this context. By this time, it was clear that the progresses made in genomic selection (genomic tools, number of markers, genomic knowledge from parents/former generations, precision of the genetic estimation related to the size of reference populations) increased the potential advantages of using embryo genotyping. The technique is now used by the major breeding companies in Europe and worldwide ( Le Bourhis *et al*., 2010 ; Shojaei Saadi *et al*., 2014 ). Two years ago, the workshop organized on this topic (Association of Embryo Technology in Europe - AETE, 2016 ) revealed that the major limitations were related to logistics i.e access to a typing center and delay of response. This technique is also easier to handle for breeding companies running field stations with OPU donors and recipients. In this context, the percentage of genotyped embryos is no more marginal, and reach today 40% of the total number of transfer performed by some selection units in Europe (S Lacaze, 2018; Auriva, Denguin, France; personal communication, AETE activity statistics 2017). Other benefits results from the systematic eradication of known genetic defects at an early stage. In the future, embryo genotyping may favours also the management of genetic variability through the optimization of the use of available recipients which is still a limiting factor in European conditions.

There are many discussions today about emerging technologies susceptible to change completely the practices in genetic selection. Using methods derived from rodents ( Brinster and Avarbock, 1994 ), advances in culturing cattle and pig spermatogonial stem cells (SSCs) have occurred over the past few years ( Oatley, 2018 ). These cells have the capacity to regenerate spermatogenesis following transplantation into testes of a recipient male that lacks endogenous germline. There is still limitations in the proliferation of SSCs to provide sufficient numbers of cells for transfer into multiple recipient males. If successful, this ability could be exploited in livestock production as a breeding tool to shorten generation interval then enhancing genetic gain. Another possibility raised from the recent work of Bogliotti *et al*. (2018) would be to use embryonic stem cells as donors for nuclear transfer to produce blastocysts. Both types of techniques could be combined with gene editing to produce animals with close specific characteristics. The potential interest of Parental Allele Gene Editing (PAGE) for selection has been put forward a few years ago ( Jenko *et al*., 2015 ). On the contrary, recent reports showed that the genetic gain allowed by gene editing would be quite low (especially if causal mutations corresponding to a given trait are not perfectly identified) and extremely costly ( Simianer *et al*., 2018 ). It may be possible to overcome all technical issues one day or another. However it is very difficult to see how the intensive production from a limited set of donor animals induced by these technologies would not be detrimental to genetic variability. As its maintenance in the main dairy breeds is of a crucial importance today to insure the sustainability of dairy cattle productions ( Colleau and Sargolzaei, 2011 ; Colleau *et al*., 2017 ; V. Ducrocq, 2018; INRA, Jouy en Josas, France; personal communication), it is unlikely, due also to the limitations of PAGE in the context of complex traits ( Gao et al., 2017 ), that use of these new technologies will develop quickly in dairy cattle selection schemes. Nevertheless, it will be interesting to follow the development of these techniques and the place they may find for other types of productions and in other species. The social acceptability of these techniques should also be discussed in the future when considering the growing concern related to a more natural approach of breeding practices.

## Functional genomics in reproductive tissues

### Relationships between reproductive phenotypes and gene expression

By the end of the 90’s, methods based on use of a limited set of informative genomic regions (initially a few QTLs, quantitative trait loci) were implemented in selection schemes ( Meuwissen and Goddard, 1999 ). The promising results obtained in terms of genetic progress raised the need to enrich and refine the set of markers available. An agreement between breeding companies and the French national research funding agencies created a favourable environment to run genomic studies aiming at developing new methods and at identifying new markers for genomic selection. As other functional traits, reproductive traits were among the most difficult to select with conventional methods and thus were susceptible to benefit largely from genomic selection. This gave us the opportunity to initiate projects to relate phenotypic and genomic information in reproductive tissues. A QTL approach based on the registration of precise phenotypic information obtained in young bulls allowed the identification of 15 markers for sperm quality ( Druet *et al*., 2009 ). As early embryonic mortality or lack of fertilization were major sources of poor fertility (see above #1) and due to the strong relationships existing between oocyte growth, maturation, the first cleavages and the success of subsequent embryonic development and maintenance of pregnancy ( Lonergan *et al*., 1999 ; Sirard, 2001 ; Sirard *et al*., 2006 ; Lequarré *et al*., 2005; Humblot *et al*., 2005 ), several projects aimed at studying oocyte quality and related gene expression ( Pennetier *et al*., 2005 ). Putative markers were identified from extreme phenotypes (Guyader Joly *et al *., 2007) and differential gene expression in relation with oocyte maturation ( Angulo *et al*., 2015 ). Benefits have been taken also from the experience obtained from the study of the sources of variation of reproductive performance ( Grimard *et al*., 2006 ) to approach differences in fertility between progeny groups from a large data base and relate them to the existence of candidate mutations in Holstein cows ( Ledoux *et al*., 2015 ). This work allowed the identification of one QTL for early embryonic mortality in the Prim’Holstein cow (Lefebre *et al.*, 2011).

The results from these first functional studies on reproductive genomics had so far a marginal impact on genomic selection progressing mainly today from the use of a very large set of markers with whole genome approaches (V. Ducrocq, 2018; INRA, Jouy en Josas, France; personal communication). However, these projects helped to clarify the specific impacts of genetic variants on reproductive function ( Coyral-Castel *et al*., 2011 ; Ledoux *et al*., 2015 ). In other projects, attention was paid on the relationships between reproduction and metabolism. The ability of cows to be fertilized and sustain pregnancy was investigated through the study of the effects of diet on gene expression in the genital tract ( Valour *et al*., 2013 ). As a continuation, we use the cow endometrium and *in vitro* models to study the impacts of metabolic and infectious stress on gene expression and pro-inflammatory response in the endometrium ( Chanrot *et al*., 2017a , b ; Guo *et al*., 2016 ; Piras *et al*., 2017 ; Chankeaw *et al*., 2018 ). Together with others ( Oguejiofor *et al*., 2015a , b ; Salilew-Wondim *et al*., 2016 ), these studies reveal that infectious stress alters a very large number of genes belonging to pro-inflammatory, proliferative, metabolic and oxidative stress (over-expressed) and to cell structure and cell adhesion (under-expressed) pathways. Some of these changes have been documented in different cow models, but the above studies show that alterations of endometrial function concerns also a large number of genes involved specifically in maternal recognition of pregnancy ( Cheng *et al*., 2017 ), immune-tolerance and implantation ( Guo *et al*., 2016 ; Piras *et al*., 2017 ). Such studies bringing a more complete view of alterations induced by pathogens, pave the way for *in vivo* work and will probably be the source of alternative therapies in the future.

In addition, some of the above studies confirm the links between metabolic imbalance and increased sensitivity to infectious stress through increased gene expression promoting pro-inflammatory reactions. Together with other approaches based on metabolomics (Munoz *et al* ., 2014a, b) measurement of NEFA’s in milk ( Martin *et al*., 2015 ) or other biomarkers ( Adnane *et al*., 2017 ) they may allow developing predictive tools to evaluate the ability of cows to re-establish ovarian activity, be able to sustain pregnancy following AI or embryo transfer and increase success rates.

### The “renaissance” of Epigenetics and its potential for selection and precision medicine

Different theories have been put forward with an evolutionary perspective. Among those the theory of Lamark (1744-1829) proposed a “soft adaptation” of species to their environment and possible transmission of induced changes to next generations. This theory has been debated for long but has taken over former criticism with the accumulation of scientific evidence obtained from examples showing the importance of transgenerational epigenetics ( Haig, 2007 ). The concept that gene expression is controlled by epigenetic mechanisms and that DNA associated molecular patterns can be transferred to next generations is now well established ( Segars and Aagaard-Tillery, 2009 ). The knowledge accumulated in that field based on the development of next generation sequencing technologies raises numerous common challenges to be addressed for public health and animal health. Humans and animals share a large number of diseases, for instance either metabolic diseases or those induced by pathogens. The occurrence and severity of diseases are often determined by the environment humans and animals are exposed to. There is now evidence that the development/severity of many diseases is linked to epigenetic mechanisms controlling for instance DNA accessibility to pathogens and improper immune response of host ( Doherty *et al*., 2016 ). The fact that some of the mechanisms initiating the development of metabolic, cardio-vascular or neurological diseases are taking place during the peri-conception period is now largely documented ( Van Soom and Fazeli, 2015 ; Fazeli and Holt, 2017 ; Ord *et al*., 2017 ). This put the maternal environment and more generally reproduction in a central place and associated knowledge particularly critical for public and animal health. The involvement of epigenetic mechanisms in the development of diseases represents already a huge field of research in the human species. Animals are intensively used as experimental models or often as sentinels in the case of wild life to evaluate the impact of the environment on diseases ( Guillette *et al*., 2016 ). However, there are many fields where specific research made in animals (including livestock species), can contribute to improve animal productions and welfare. Progresses made in the description of animal genomes and reduction of the costs, offers new opportunities to perform these studies and it can be foreseen that epigenetic studies will be the basis for new developments for reproductive physiology and biotechnologies. Obtained from models or taken from the environment, the identification of extreme phenotypes combined with use of genetic and epigenetic information allowed already the identification of critical set of genes and epigenetic marks associated to cell processes as altered responses to disease ( Jhamat *et al*., 2016 ). Although more complex such links can be established between the embryo and its environment either metabolic ( Laskowski *et al*., 2018 ) and or induced by use of embryo technologies like cloning ( Beaujean, 2014 ; Sepulveda-Rincon *et al*., 2016 ) or embryo culture ( Salvaing *et al*., 2016 ). Further steps include functional studies to validate the differences observed at the genetic and epigenetic levels and eventually the role of critical genetic variants that would be associated with increased risk to suffer from diseases. Due to the complexity of the mechanisms involved, especially the multiplicity of epigenetic marks and their respective roles, it will take time to integrate epigenetics in selection schemes but its association with genomic information can be anticipated ( Britt *et al*., 2018 ; V. Ducrocq, 2018; INRA. Jouy en Josas, France; personal communication) especially in the field of resistance to disease. There are also emerging fields such as inter-cellular communication through extra-cellular vesicles ( Hwang, 2013 ). New knowledge about the molecular and especially the epigenetic signals they vehicle, will bring substantial improvements in the diagnostic and therapy of diseases ( Giebel *et al*., 2017 ) and probably be the source of critical information for embryo survival ( Rizos *et al*., 2017 ).

## Conclusions

This retrospective and present developments in genetics biology, developmental biology and reproductive techniques raise a lot of questions in many different fields. It is not possible at the moment to give the right answers for most of them. However there is no doubt that these questions are very critical for future generations especially in Livestock species due to the weight of human decisions and the acceleration in the selection process associated to genomic selection. Reproduction and reproductive technologies will go on to play a central role as their efficiency is a key element to preserve genetic variability. New technologies such as genome editing or reproduction techniques based on germinal cells of a few individuals to shorten generation interval are tempting for the breeding industry. On top of technical limitations and high costs, there is a big risk that they will increase dramatically consanguinity thus altering many other traits such as reproductive efficiency. It is not risky to predict that phenotyping will go on to progress and this will be very positive to facilitate the selection for new traits important for animal health and or the environment. As part of the picture, although still rather complex to obtain due to limitations of the bovine genome, the integration of epigenetic data in selection schemes is promising especially for traits such as resistance to diseases. Reproduction and especially embryo developmental biology stands in the central place to approach the epigenetic mechanisms translating the impact of the environment on individuals especially at time of peri-conception which induce the development of physio-pathological processes leading to diseases. Strong limitations still exist when approaching for instance reproductive diseases or the impact of the environment on reproduction from molecular studies generating huge amounts of data to be integrated. The end of this reproductive journey revealed the need to reinforce the tiny links between the communities of reproductive physiologists, molecular geneticists, bio-informaticians and bio-statisticians to investigate arising problems with common translational approaches.
